# Longitudinal versus cross-sectional studies of effects of aging on ventricular structure and function using cardiac magnetic resonance imaging

**DOI:** 10.1186/1532-429X-11-S1-P4

**Published:** 2009-01-28

**Authors:** Dipti Gupta, Mark Goldman, Sunil T Mathew, Jing Han, William Schapiro, Michael Passick, Katherine McGrath, Jie J Cao, Nathaniel Reichek

**Affiliations:** 1 St. Francis Hospital, Roslyn, NY USA; 2 University of Oklahoma, Oklahoma city, OK USA

**Keywords:** Body Surface Area, Cardiac Magnetic Resonance, Cardiac Magnetic Resonance Imaging, Aging Effect, Axis Slice

## Introduction

Cross-sectional studies of effects of aging on ventricular structure and function, including Framingham, MESA, the Dallas Heart Study and studies from our own laboratory have uniformly demonstrated lower ventricular chamber volumes in older normal subjects.

## Purpose

We sought to assess aging effects on ventricular size and function prospectively in a carefully screened normal cohort.

## Methods

Normotensive, non-diabetic, non-obese (BMI < 28) volunteers (n = 57, 31 females), aged 20–89 at intake (59 ± 13 yrs), were screened, including 2D echocardiography and CMR performed (1.5 T Siemens Sonata or Avanto) at baseline and 5 years. TrueFISP cine imaging was used to obtain contiguous 8 mm short axis slices of both left (LV) and right (RV) ventricles. LV and RV volumes at end-diastole and end-systole were determined (Medis, MASS) and indexed (i) to body surface area. Ejection fraction (EF) was also calculated.

## Results

Systolic blood pressure increased (120 ± 12 to 130 ± 20 mm Hg, p < .0001), LV and RV end diastolic volumes increased (113 ± 30 to 136 ± 37, 114 ± 31 to 134 ± 37 ml, p < .0001 for both), as did end systolic volumes (45 ± 17 to 59 ± 23, 55 ± 21 to 60 ± 22 ml, p < .0001 and p = .01 respectively). LVEF decreased (61 ± 7 to 58 ± 7%, p < .004), while RVEF increased slightly (53 ± 6 to 56 ± 7%, p = .01). There were no major gender differences. Subjects with intake age <50 years and those ≥50 years behaved similarly.

## Conclusion

In contrast to cross-sectional studies, which demonstrate reduced LV and RV volumes in older subjects, this prospective study demonstrates that chamber volumes increase with age. This discordance may be due to generational differences which affect cross-sectional, but not longitudinal studies.

